# Survival analysis of intraoperative blood salvage for patients with malignancy disease: A PRISMA-compliant systematic review and meta-analysis

**DOI:** 10.1097/MD.0000000000016040

**Published:** 2019-07-05

**Authors:** Wei-Wei Wu, Wei-Yi Zhang, Wei-Han Zhang, Lei Yang, Xiao-Qian Deng, Meng-Chan Ou, Yao-Xin Yang, Hai-Bei Liu, Tao Zhu

**Affiliations:** a Department of Anesthesiology, West China Hospital; b Department of Gastrointestinal Surgery and Laboratory of Gastric Cancer, State Key Laboratory of Biotherapy, West China Hospital, Collaborative Innovation Center for Biotherapy, Sichuan University, Chengdu, Sichuan, China.

**Keywords:** allogenic blood transfusion, intraoperative blood salvage, recurrence rate, survival outcomes, tumor

## Abstract

**Background:**

Intraoperative blood salvage as a blood-saving strategy has been widely used in surgery. Considering its theoretic risk of malignant tumor cells being reinfused and the corresponding blood metastases, the safety of intraoperative blood salvage in cancer surgery remains controversial.

**Methods:**

Following the Preferred Reporting Items for Systemic Review and Meta-Analysis (PRISMA), we searched the Cochrane Library, MEDLINE and EMBASE to November 2017. We included only studies comparing intraoperative blood salvage with allogeneic blood transfusion.

**Results:**

This meta-analysis included 9 studies with 4354 patients with 1346 patients in the intraoperative blood salvage group and 3008 patients in the allogeneic blood transfusion group. There were no significant differences in the 5-year overall survival outcome (odds ratio [OR] 1.12; 95% confidence interval [CI], 0.80–1.58), 5-year disease-free survival outcome (OR 1.08; 95% CI 0.86–1.35), or 5-year recurrence rate (OR 0.86; 95% CI 0.71–1.05) between the 2 study groups. Subgroup analysis also showed no significant differences in the 5-year overall survival outcome (OR 0.97; 95% CI 0.57–1.67) of hepatocellular carcinoma patients in liver transplantation.

**Conclusions:**

For patients with malignant disease, intraoperative blood salvage did not increase the tumor recurrence rate and had comparable survival outcomes with allogeneic blood transfusion.

## 1 Introduction

Intraoperative blood salvage (IBS) is a kind of blood-saving strategy that uses autologous transfusion and is widely used during surgery with massive blood loss to reduce the allogenic blood transfusion volume.^[1,2]^ Generally, autologous transfusion includes the following 3 modalities: first, preoperative autologous blood donation (ABD), including predeposited autologous blood that was stored, retransfused during surgery, and requires patients to donate blood before ≥2 before surgery; second, acute normovolemic hemodilution (ANH), which requires collecting blood preoperatively with subsequently artificial dilute and reinfuse during surgery; third, IBS, an attractive blood management strategy, retrieves and filters blood lost during the operation and then instantly reinfuses it.^[3]^ In addition, IBS could eliminate many complications associated with storing and processing homologous donor blood.^[4]^ However, many surgeons still hesitate to embrace IBS for its theoretical risk of increasing the tumor recurrence rate.^[5]^ These surgeons presume that tumor cells would be reinfused with IBS blood by cell saver, which would result in tumor cell dissemination. Even though this hypothesis is unwarranted, the use of IBS is still restricted by this conjecture. A case report published in 1975 reported that IBS may have cause neoplasm metastasis during the operation of a lung cancer patient.^[5]^ Since then, IBS is no longer recommended for tumor-related operations.^[6]^ In view of this conjecture, whether or not tumor cells pass through the cell saver system is the major point of controversy and significantly hampers the clinical utility of IBS. With the development of materials science, the leukocyte depletion filter (LDF) has been suggested to effectively remove a variety of malignant cells in spine tumor surgery and colorectal tumor surgery.^[7]^ In addition, allogeneic transfusion is widely used in clinical practice, but it also has some inherent limitations, such as allergic reaction, infection, hemolysis, perioperative myocardial infarction, postoperative low-output cardiac failure, transfusion-related immunomodulation, transfusion-related acute lung injury, and life-threatening virus infection.^[8–16]^ Compared with allogeneic blood transfusions, IBS seems to be a better choice regardless of the effect-cost ratio or efficacy in tumor operation.^[17]^ Even though some doubts and controversies in the IBS still exist, existing evidence has indicated that it was not the major reason for tumor metastasis.^[18]^

However, some of the previous studies did not focus on pure intraoperative blood salvage, and they analyzed the other subtype of autologous transfusion methods.^[19,20]^ Therefore, we conducted this meta-analysis with the pretension to evaluate the oncological safety of pure IBS compared with allogeneic blood transfusion (ABT) in operations of malignant disease.

## 2 Methods

Following the Preferred Reporting Items for Systemic Review and Meta-Analysis (PRISMA),^[21]^ we searched the Cochrane Library (January 1, 2005–November 24, 2017), MEDLINE via PubMed (January 1, 1966–November 24, 2017), and EMBASE (January 1, 1980–November 24, 2017). We combined searching methods with free words and subject terms in searching databases. We searched the terms “Blood Transfusion, Autologous” and “Neoplasms” in PubMed and Cochrane Database of Systematic Reviews and “Blood autotransfusion” in EMBASE as subject terms. The following terms were also utilized: “cell salvage,” “cell saver,” “blood salvage,” “autotransfusion,” “autologous transfusion,” “Blood Cell Salvage,” and “retransfusion.” Because studies included in this meta-analysis have been published, it is not needed for the ethical approval from ethics committees.

After searching the databases, 2 researchers screened and excluded the articles through title and abstract according to inclusion criteria. We included only English studies that compared IBS and ABT during the operation, regardless of what research type or publication status. The intervention group was strictly confined to the IBS method. Those studies with the autotransfusion method of ABD or ANH were excluded. Therefore, the intervention group comprises patients who accepted pure IBS therapy, and the control group comprises patients with the same type of malignant tumor who used ABT in their operations. The studies that passed the first-round selection were further filtered by reading the full-text and removed from this study based on exclusion criteria by 2 researchers.

The data analyzed in this study were extracted from the full-text article and include the following parameters: name of the first author, periodical titles, publication year, type of tumor, IBS group characteristics, control group characteristics (allogeneic blood transfusion group), exclusion criteria, sample size, length of follow-up, and mean patient age (Table 1). One author extracted data, and another author checked this process. We also strived to search for any relevant information from the references of every included report.

**Table 1:**
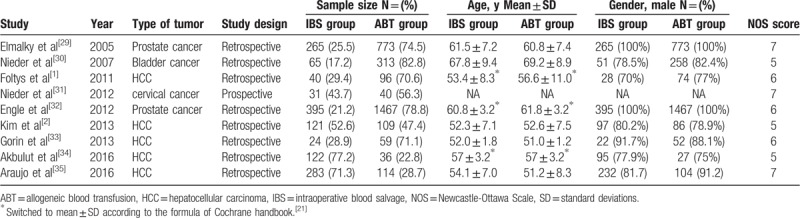
The characteristics of included studies.

The quality assessment of all of the included studies were determined by 2 authors based on the Newcastle-Ottawa Scale (NOS), and those studies had at least a score of 5.^[22]^ Therefore, we deemed that the included studies were reliable for this meta-analysis. Articles were evaluated and discussed with a third person when any divergence existed.

The primary outcome of the present study is the tumor-related recurrence rate (RR). We collected and analyzed the 1-year recurrence rate, 3-year recurrence rate, and 5-year recurrence rate. In addition, other survival outcomes, including the overall survival (OS) rate and the disease-free survival (DFS) rate, were analyzed and reported as well. Other parameters, such as the volume of allogeneic blood transfusion in the perioperative period (mL), the allogeneic blood transfusion rate in the perioperative period, and the length of hospitalization, were also collected and analyzed.

The dichotomous data were compared with odds ratio (OR) and the continuous data by the mean difference (MD). We switched the continuous data presented as median (quartile or range) to the mean (standard deviation) based on a certified formula.^[23]^ The outcomes were estimated with a random-effects model. Statistic heterogeneity was presented by both chi-squared value and *I*^2^. Publication bias was assessed by funnel plots. Sensitivity analysis was used to ensure the credibility of the result. All above analyses were processed using Review Manager 5.3 (Cochrane Collaboration, Oxford, UK) and STATA version 14.1 (Stata Corp LP, College Station, TX).

Considering the inherent heterogeneity of different kinds of tumors, we conducted subgroup analyses of the 5 studies for liver cancer surgery. We collected and evaluated the 5-year overall survival for the patients from these studies.

## 3 Results

We retrieved 3169 records within 255 duplicates. After reading the titles and abstracts, 74 articles remained for reassessing according to their full-text. After reading the full-text of these articles, we included 17 studies in which blood salvage was performed intraoperatively. However, 8 of these studies were excluded due to a lack of outcome indicators (1995, Connor et al^[18]^; 1999, Mirhashemi et al^[24]^; 2005, Stoffel et al^[25]^; 2010, Ubee et al^[26]^; 2011, Bower et al^[27]^; 2011, Ubee et al^[17]^; 2015, Lyon et al^[28]^; 2017, Elmalky et al).^[29]^ In total, only 9 studies were available to pool into this meta-analysis^[1,2,30–36]^ (PRISMA Flow Diagram).

Except for the study from Engel et al,^[32]^ which was a prospective study, all of the other included studies were retrospective studies. We also evaluated the quality of all included studies with the NOS scale, and the results showed that all studies had a score >5. Five studies reported a follow-up period as follows: 25.8 ± 15.1 months in the IBS group and 17.9 ± 12.8 months in the ABT group.

**Figure 1:**
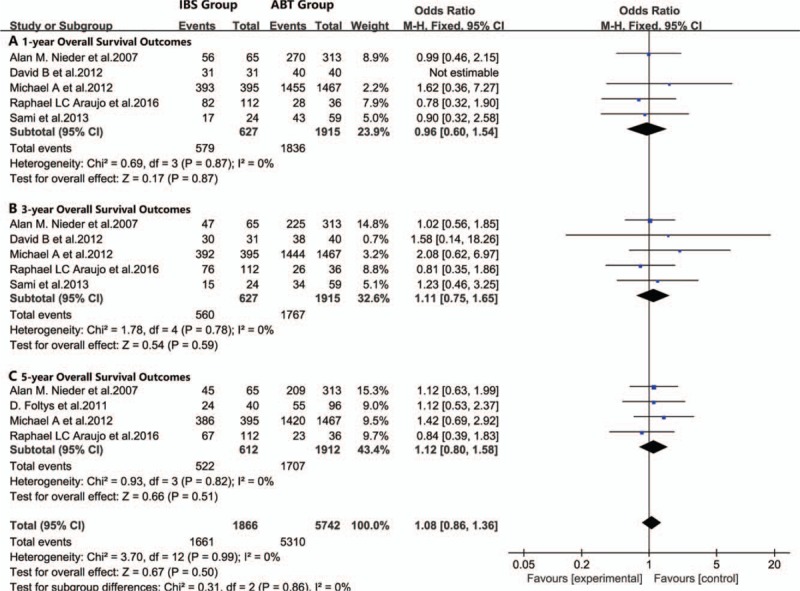
Meta-analysis forest plot of the overall survival outcomes. (A. 1-year overall survival outcome, B. 3-year overall survival outcome, C. 5-year overall survival outcomes).

**Figure 2:**
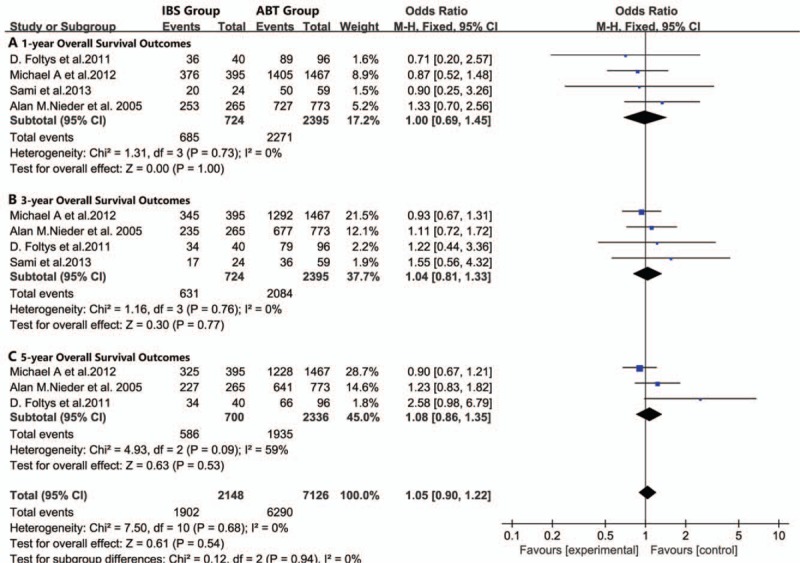
Meta-analysis forest plot of the disease-free survival outcomes. (A. 1-year disease-free survival outcome, B. 3-year disease-free survival outcome, C. 5-year disease-free survival outcomes).

**Figure 3:**
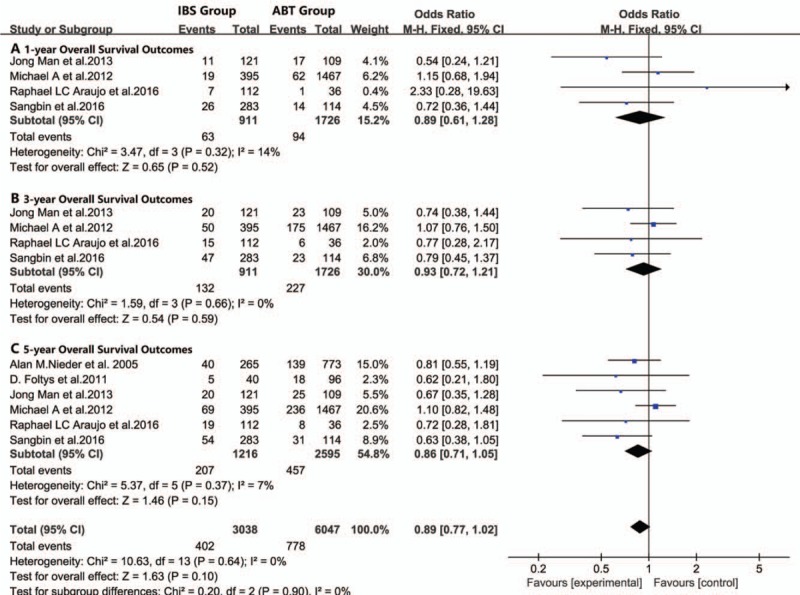
Meta-analysis forest plot of the recurrence rate. (A. 1-year recurrence rate, B. 3-year recurrence rate, C. 5-year recurrence rate).

For all included studies performed in statistics, there were no significant differences in overall survival (Fig. 1), disease-free survival (Fig. 2), or recurrence rate (Fig. 3) between patients in the IBS group or patients in the ABT group. In subgroup analyses, there were no significant differences in the postoperative 1-year recurrence rate (95% CI, 0.61–1.28; *P* = .32, Fig. 3A), 3-year recurrence rate (95% CI, 0.72–1.21; *P* = .66, Fig. 3B) or 5-year recurrence rate (95% CI, 0.71–1.05; *P* = .37, Fig. 3C) between the IBS and ABT groups. In addition, we noticed that patients in the IBS group showed a lower recurrence rate than the ABT group in 2 studies. However, these 2 studies showed similar overall survival outcomes and disease-free outcomes between the 2 transfusion methods.^[2,36]^ Publication biases were not observed in this meta-analysis, with the *P* value for the Egger linear test of 0.245 (*t* = −1.44).

All stage of grade of tumor of included studies have not reported to be difference significantly. Only 2 of them reported postoperative complications.^[2,31]^ Kim et al^[2]^ reported non-IBS group has higher renal dysfunction (*P* = .028), bleeding (*P* = .046), bacterial infection (*P* = .012), and urinary tract infection (*P* < .001) morbidity.

There was no noticeable heterogeneity between the IBS and ABT groups in overall survival (*I*^2^ = 0%, *P* = .99), disease-free survival (*I*^2^ = 0%, *P* = .68), or recurrence rate (*I*^2^ = 0%, *P* = .64).

**Figure 4:**
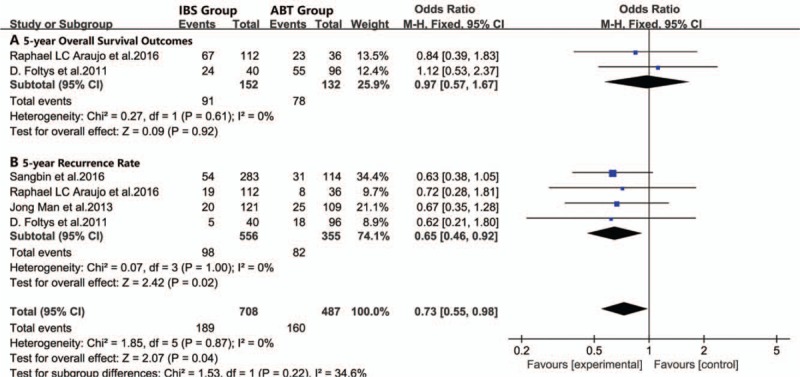
Meta-analysis forest plot of survival outcomes of hepatocellular carcinoma patients (A. 5-year overall survival outcomes, B. 5-year recurrence rate).

In addition, we considered the potential bias associated with different diseases and operations. A subgroup analysis was performed on 5 studies, which focused on liver transplantation surgery,^[1,2,34–36]^ and the results showed that there were also no significant differences in the 5-year overall survival outcomes (95% CI 0.57–1.67, *P* = .92, Fig. 4A) between the IBS and ABT groups. Remarkably, the IBS group showed a lower 5-year overall recurrence rate than the ABT group (95% CI, 0.46–0.92, *P* = .02, Fig. 4B). Both of these studies presented low heterogeneity (*I*^2^ = 0%) in the overall survival and recurrence rate.

## 4 Discussion

In the present study, we used the recurrence rate as the primary outcome, and the overall survival and disease-free survival was used as the secondary outcomes. In total, 6 included studies performed overall survival as the primary outcome, and 4 included studies performed disease-free survival as the primary outcome. There were no significant differences between the IBS and ABT groups in the 1-year, 3-year, or 5-year overall survival or disease-free survival outcomes. Meanwhile, for the 6 included studies, patients in the IBS group had a similar recurrence rate as patients in the ABT group.

A study reported that the average intraoperative blood loss in open radical retropubic prostatectomy is over 1000 mL.^[17]^ In addition, a report based on 984 living donors presented that the mean intraoperative blood loss in hepatic resection was 691.3 ± 365.5 mL.^[37]^ Given these results and considering the potential intraoperative blood loss in surgery, surgeons should always be prepared for preoperative transfusion. However, allogenic blood transfusions were associated with various complications that threatened patient recovery and prolonged hospital stays. Even so, the thought of the potential risk of tumor cells being collected intraoperatively along with blood and then reinfused into patients that may result in tumor metastasis, most surgeons do not take IBS into account. However, there are no large-sample multicenter random control trials to support this thesis and is based on a case report in 1975 that reported that tumor cells were found in the cell saver.^[5]^ This finding directly resulted in the American Medical Association Council on Scientific Affairs stopping intraoperative autologous transfusion used for cancer surgery.^[6]^

Nowadays, IBS has been proven that it could reduce postoperative complications and has shown to be cost-effective.^[2]^ However, whether intraoperative autologous transfusion truly increases the risk of tumor metastasis remains controversial. Great efforts have been made to prove this technique to be efficient and safe. In the present study, we found no significant differences between the IBS and ABT groups in overall survival outcomes, disease-free outcomes, or recurrence rates. These results are consistent in the 1-year, 3-year, and 5-year subgroup analyses. However, we must emphasize that this result is based on different kind of tumor studies. Additionally, this meta-analysis included only a few kinds of malignant diseases, such as hepatocellular carcinoma and urogenital tumors. On the one hand, considering the tumor heterogeneity, the metastasis risk is completely different in different kinds and stages of tumors.^[38–40]^ Considering the variety of malignant diseases, the results of these analyses may have selection bias. On the other hand, there was only one prospective study, and other included studies were retrospective studies. The natural limitation of retrospective studies cannot be neglected.

We noticed that 5 studies focused on hepatocellular carcinoma. Therefore, we performed a subgroup analysis comparing the 5-year overall survival outcomes between the IBS and ABT groups. Compared with ABT, IBS did not improve the mortality risk with long-term follow-up for patients with hepatocellular carcinoma who underwent liver transplantation surgery. Interestingly, we found that the 5-year recurrence rate in the IBS group was significantly lower than that in the ABT group. This result may be because transfusion-related immune modulation would accelerate cancer progression. Two meta-analyses have shown that ABT is associated with postoperative survival in colorectal cancer and carcinoma of the duodenal ampullary.^[41–43]^ Even though we need more evidence in the other types of cancer surgery; however, more studies may imply that we should strive to reduce the intraoperative ABT. However, limited numbers of studies were included in these analyses. This phenomenon illustrates that IBS is not inferior to allogeneic blood transfusion and may even be better than ABT.

Circulating tumor cells may be the key factor that results in distal metastasis of the tumor. The leukocyte filter was proven to filter the hemangiosarcoma and hepatocellular carcinoma cells completely in experiments.^[44,45]^ Meanwhile, Kumar et al^[46]^ found that intraoperative cell salvage with a leucocyte filter can effectively eliminate tumor cells from salvaged blood in spinal tumor surgery. However, whether tumor cells are completely filtered in clinical settings and whether the filter eliminates the risk of tumor cell metastasis are still pending. However, with the development of technology, the combined use of the new generation leukocyte filter may be the hope for the widespread promotion of IBS.

In addition, several studies have successfully confirmed that the autologous transfusion strategy can reduce the need for allogeneic blood during an operation.^[1,2]^ However, these conclusions were made by comparing all 3 subtype methods of autologous transfusion and allogeneic blood transfusion.^[19,20]^ Current evidence supports the idea that IBS could reduce the need for a blood transfusion. Only 4 included studies compared the allogeneic blood transfusion volume between 2 groups, but the heterogeneity was high (*I*^2^ = 85%). Therefore, it is difficult to conclude that the IBS can save the amount of allogeneic blood.

To evaluate the safety and efficiency of IBS, this meta-analysis included 9 studies and showed that IBS was comparable with ABT. However, several limitations are also included in the present study, as follows: we only included studies that compared IBS with ABT, and most of the included studies were retrospective research; the selection bias cannot be neglected. The study included several malignant diseases, and the natural difference between these tumors may affect the prognosis of patients; the hybrid effect brought by the retrospective study and different kinds of tumors may lead to a bias of the final results. Therefore, a further large-sample size randomized control study with each kind of tumor surgery is expected to solve these limitations.

## 5 Conclusions

During surgery for malignant tumors, intraoperative blood salvage did not increase the tumor recurrence rate and had comparable survival outcomes with allogeneic blood transfusion. However, due to the limitation of evidence, the wide application of intraoperative blood salvage requires further multicenter randomized control trials to verify these results.

## Acknowledgments

The authors thank the Chinese Evidence-based Medicine Center West China Hospital, Sichuan University for providing statistical consultation.

## Author contributions

Wei-Wei Wu and Wei-Yi Zhang conceived the project with input from Tao Zhu.

Wei-Wei Wu and Wei-Yi Zhang designed the study and wrote the protocol with the input from all authors.

Wei-Wei Wu and Wei-Yi Zhang did the literature searches and processed the trial data.

Wei-Wei Wu, Wei-Yi Zhang, Wei-Han Zhang designed the statistical analyses, and Wei-Wei Wu, Lei Yang, and Xiao-Qian Deng performed the statistical analyses.

Wei-Wei Wu and Wei-Yi Zhang drafted the manuscript.

All authors have seen and commented on the drafts and approved the final version.

**Conceptualization:** Wei-Wei Wu, Wei-Yi Zhang, Tao Zhu.

**Data curation:** Wei-Yi Zhang.

**Formal analysis:** Wei-Yi Zhang, Wei-Han Zhang.

**Funding acquisition:** Wei-Han Zhang, Xiao-Qian Deng.

**Methodology:** Wei-Wei Wu, Wei-Han Zhang, Lei Yang, Meng-Chan Ou, Yao-Xin Yang.

**Resources:** Tao Zhu.

**Software:** Wei-Wei Wu, Lei Yang.

**Supervision:** Wei-Yi Zhang, Hai-Bei Liu.

**Writing – original draft:** Wei-Wei Wu, Wei-Yi Zhang.

**Writing – review & editing:** Wei-Wei Wu, Wei-Yi Zhang, Wei-Han Zhang, Lei Yang, Xiao-Qian Deng, Meng-Chan Ou, Yao-Xin Yang, Hai-Bei Liu, Tao Zhu.

## References

[1] Foltys D Zimmermann T Heise M Liver transplantation for hepatocellular carcinoma--is there a risk of recurrence caused by intraoperative blood salvage autotransfusion? Eur Surg Res 2011;47:182–7. 21986299 10.1159/000330746

[2] Kim JM Kim GS Joh JW Long-term results for living donor liver transplant recipients with hepatocellular carcinoma using intraoperative blood salvage with leukocyte depletion filter. Transpl Int 2013;26:84–9. 23194351 10.1111/tri.12001

[3] Brzica SM Jr Pineda AA Taswell HF Autologous blood transfusion. Mayo Clin Proc 1976;51:723–37. 994552

[4] Basran S Frumento RJ Cohen A The association between duration of storage of transfused red blood cells and morbidity and mortality after reoperative cardiac surgery. Anesth Analg 2006;103:15–20. table of contents. 16790618 10.1213/01.ane.0000221167.58135.3d

[5] Yaw PB Sentany M Link WJ Tumor cells carried through autotransfusion. Contraindication to intraoperative blood recovery? JAMA 1975;231:490–1. 1172829

[6] Autologous blood transfusions. Council on scientific affairs. JAMA 1986;256:2378–80. 3773142

[7] Kumar N Lam R Zaw AS Flow cytometric evaluation of the safety of intraoperative salvaged blood filtered with leucocyte depletion filter in spine tumour surgery. Ann Surg Oncol 2014;21:4330–5. 25069862 10.1245/s10434-014-3950-9

[8] Whitlock EL Kim H Auerbach AD Harms associated with single unit perioperative transfusion: retrospective population based analysis. BMJ 2015;350:h3037. 26070979 10.1136/bmj.h3037 PMC4463965

[9] Vamvakas EC Possible mechanisms of allogeneic blood transfusion-associated postoperative infection. Transfus Med Rev 2002;16:144–60. 11941576 10.1053/tmrv.2002.31463

[10] Ueda J Ikota N Hanaki A Synthesis of new oligopeptides and their scavenging abilities against active oxygen species. Biochem Mol Biol Int 1994;33:1041–8. 7804128

[11] Flohe S Kobbe P Nast-Kolb D Immunological reactions secondary to blood transfusion. Injury 2007;38:1405–8. 18048035 10.1016/j.injury.2007.09.028

[12] Goubran H Sheridan D Radosevic J Transfusion-related immunomodulation and cancer. Transfus Apher Sci 2017;56:336–40. 28606449 10.1016/j.transci.2017.05.019

[13] Silliman CC McLaughlin NJ Transfusion-related acute lung injury. Blood Rev 2006;20:139–59. 16360246 10.1016/j.blre.2005.11.001

[14] Sachs UJ The pathogenesis of transfusion-related acute lung injury and how to avoid this serious adverse reaction of transfusion. Transfus Apher Sci 2007;37:273–82. 18036987 10.1016/j.transci.2007.02.005

[15] Asmuth DM Kalish LA Laycock ME Absence of HBV and HCV, HTLV-I and -II, and human herpes virus-8 activation after allogeneic RBC transfusion in patients with advanced HIV-1 infection. Transfusion 2003;43:451–8. 12662277 10.1046/j.1537-2995.2003.00350.x

[16] Goodnough LT Brecher ME Kanter MH Transfusion medicine. First of two parts--blood transfusion. N Engl J Med 1999;340:438–47. 9971869 10.1056/NEJM199902113400606

[17] Ubee S Kumar M Athmanathan N Intraoperative red blood cell salvage and autologous transfusion during open radical retropubic prostatectomy: a cost-benefit analysis. Ann R Coll Surg Engl 2011;93:157–61. 22041147 10.1308/003588411X561044 PMC3293313

[18] Connor JP Morris PC Alagoz T Intraoperative autologous blood collection and autotransfusion in the surgical management of early cancers of the uterine cervix. Obstet Gynecol 1995;86:373–8. 7651645 10.1016/0029-7844(95)00183-R

[19] Williamson KR Taswell HF Intraoperative blood salvage: a review. Transfusion 1991;31:662–75. 1891796 10.1046/j.1537-2995.1991.31791368347.x

[20] Zaw AS Bangalore Kantharajanna S Kumar N Is autologous salvaged blood a viable option for patient blood management in oncologic surgery? Transfus Med Rev 2017;31:56–61. 27421661 10.1016/j.tmrv.2016.06.003

[21] Moher D Liberati A Tetzlaff J Group P. Preferred reporting items for systematic reviews and meta-analyses: the PRISMA statement. PLoS Med 2009;6:e1000097. 19621072 10.1371/journal.pmed.1000097 PMC2707599

[22] Stang A Critical evaluation of the Newcastle-Ottawa scale for the assessment of the quality of nonrandomized studies in meta-analyses. Eur J Epidemiol 2010;25:603–5. 20652370 10.1007/s10654-010-9491-z

[23] Hozo SP Djulbegovic B Hozo I Estimating the mean and variance from the median, range, and the size of a sample. BMC Med Res Methodol 2005;5:13. 15840177 10.1186/1471-2288-5-13 PMC1097734

[24] Mirhashemi R Ganjei-Azar P Nadji M Papillary squamous cell carcinoma of the uterine cervix: an immunophenotypic appraisal of 12 cases. Gynecol Oncol 2003;90:657–61. 13678741 10.1016/s0090-8258(03)00329-9

[25] Stoffel JT Topjian L Libertino JA Analysis of peripheral blood for prostate cells after autologous transfusion given during radical prostatectomy. BJU Int 2005;96:313–5. 16042720 10.1111/j.1464-410X.2005.05621.x

[26] Ubee SS Manikandan R Gudimetla AR Cost benefits of intraoperative cell salvage in radical cystectomy. Indian J Urol 2010;26:196–9. 20877596 10.4103/0970-1591.65386 PMC2938542

[27] Bower MR Ellis SF Scoggins CR Phase II comparison study of intraoperative autotransfusion for major oncologic procedures. Ann Surg Oncol 2011;18:166–73. 21222043 10.1245/s10434-010-1228-4

[28] Lyon TD Ferroni MC Turner RM 2nd Short-term outcomes of intraoperative cell saver transfusion during open partial nephrectomy. Urology 2015;86:1153–8. 26387849 10.1016/j.urology.2015.09.008

[29] Elmalky M Yasin N Rodrigues-Pinto R The safety, efficacy, and cost-effectiveness of intraoperative cell salvage in metastatic spine tumor surgery. Spine J 2017;17:977–82. 28323241 10.1016/j.spinee.2017.03.004

[30] Nieder AM Carmack AJ Sved PD Intraoperative cell salvage during radical prostatectomy is not associated with greater biochemical recurrence rate. Urology 2005;65:730–4. 15833517 10.1016/j.urology.2004.10.062

[31] Nieder AM Manoharan M Yang Y Intraoperative cell salvage during radical cystectomy does not affect long-term survival. Urology 2007;69:881–4. 17482926 10.1016/j.urology.2007.01.060

[32] Engle DB Connor JP Morris PC Intraoperative autologous blood transfusion use during radical hysterectomy for cervical cancer: long-term follow-up of a prospective trial. Arch Gynecol Obstet 2012;286:717–21. 22569711 10.1007/s00404-012-2351-1

[33] Gorin MA Eldefrawy A Manoharan M Oncologic outcomes following radical prostatectomy with intraoperative cell salvage. World J Urol 2012;30:379–83. 21847657 10.1007/s00345-011-0746-4

[34] Akbulut S Kayaalp C Yilmaz M Effect of autotransfusion system on tumor recurrence and survival in hepatocellular carcinoma patients. World J Gastroenterol 2013;19:1625–31. 23538988 10.3748/wjg.v19.i10.1625 PMC3602480

[35] Araujo RL Pantanali CA Haddad L Does autologous blood transfusion during liver transplantation for hepatocellular carcinoma increase risk of recurrence? World J Gastrointest Surg 2016;8:161–8. 26981190 10.4240/wjgs.v8.i2.161 PMC4770170

[36] Han S Kim G Ko JS Safety of the use of blood salvage and autotransfusion during liver transplantation for hepatocellular carcinoma. Ann Surg 2016;264:339–43. 26501715 10.1097/SLA.0000000000001486

[37] Kim YK Chin JH Kang SJ Association between central venous pressure and blood loss during hepatic resection in 984 living donors. Acta Anaesthesiol Scand 2009;53:601–6. 19419353 10.1111/j.1399-6576.2009.01920.x

[38] Shu Y Zhang W Hou Q Prognostic significance of frequent CLDN18-ARHGAP26/6 fusion in gastric signet-ring cell cancer. Nat Commun 2018;9:2447. 29961079 10.1038/s41467-018-04907-0 PMC6026495

[39] Tohme S Simmons RL Tsung A Surgery for cancer: a trigger for metastases. Cancer Res 2017;77:1548–52. 28330928 10.1158/0008-5472.CAN-16-1536 PMC5380551

[40] Lee BM Cata JP Impact of anesthesia on cancer recurrence. Rev Esp Anestesiol Reanim 2015;62:570–5. 26026503 10.1016/j.redar.2015.04.003

[41] Cata JP Wang H Gottumukkala V Inflammatory response, immunosuppression, and cancer recurrence after perioperative blood transfusions. Br J Anaesth 2013;110:690–701. 23599512 10.1093/bja/aet068 PMC3630286

[42] Chung M Steinmetz OK Gordon PH Perioperative blood transfusion and outcome after resection for colorectal carcinoma. Br J Surg 1993;80:427–32. 8192723 10.1002/bjs.1800800407

[43] Yao HS Wang Q Wang WJ Intraoperative allogeneic red blood cell transfusion in ampullary cancer outcome after curative pancreatoduodenectomy: a clinical study and meta-analysis. World J Surg 2008;32:2038–46. 18584239 10.1007/s00268-008-9675-9

[44] Gwak MS Lee KW Kim SY Can a leukocyte depletion filter (LDF) reduce the risk of reintroduction of hepatocellular carcinoma cells? Liver Transpl 2005;11:331–5. 15719385 10.1002/lt.20346

[45] Ciepluch B Wilson-Robles H Levine G Removal of hemangiosarcoma cells from canine blood with a cell salvage system and leukocyte reduction filter. Vet Surg 2018;47:293–301. 29247544 10.1111/vsu.12760

[46] Kumar N Ahmed Q Lee VK Can there be a place for intraoperative salvaged blood in spine tumor surgery? Ann Surg Oncol 2014;21:2436–43. 24566859 10.1245/s10434-014-3569-x

